# Beta-blocker therapy does not reduce ascending aorta wall shear stress in patients with bicuspid aortic valve

**DOI:** 10.1186/1532-429X-17-S1-P399

**Published:** 2015-02-03

**Authors:** Bradley D Allen, Michael Markl, Alex J Barker, Pim van Ooij, James C Carr, S C Malaisrie, Robert O Bonow, Preeti Kansal

**Affiliations:** 1 Radiology, Northwestern University, Chicago, IL, USA; 2 Biomedical Engineering, Northwestern University, Chicago, IL, USA; 3 Cardiac Sugery, Northwestern University, Chicago, IL, USA; 4 Medicine-Cardiology, Northwestern University, Chicago, IL, USA

## Background

Ascending aorta (AAo) wall shear stress (WSS) may drive aorta dilatation in patients with bicuspid aortic valve (BAV) and β-blockers are first line medical therapy to slow this process. This study sought to determine if β-blocker therapy reduces AAo WSS in BAV patients.

## Methods

Right-left coronary leaflet fusion BAV patients on β-blockers (BB+) (n = 30, M:F = 23:7, age: 46 ± 14 years) and not on β-blockers (BB-) (n=30, M:F = 23:7, age: 46 ± 13 years) and healthy controls (n=15, age:43±11 years) underwent time-resolved, 3D phase contrast (4D flow) MRI. Patient groups were matched by systolic blood pressure (SBP), degree of aortic stenosis (AS), and AAo diameter (3.9 ± 0.7 vs. 3.9 ± 0.6 cm, p = 0.70). A 3D segmentation of the thoracic aorta was performed (MIMICS, Materlise, Belgium). Systolic 3D WSS was calculated in the thoracic aorta from 4D flow velocity acquisition and a sagittal maximum intensity projection (MIP) of WSS was generated. Systole was defined as five cardiac time frames centered at the time frame with maximum average aorta velocity. A region of interest was drawn on the MIP from the sinus of Valsalva to the brachiocephalic artery to define the AAo, and this region was further subdivided into anterior and posterior segments. Max and mean systolic AAo WSS were extracted from each segment. Peak systolic AAo velocity was also measured. Quantitative results were compared with one-way analysis of variance and linear modeling was performed.

## Results

Maximum and mean WSS were not reduced in the BB+ group compared BB- patients in either the anterior AAo (maximum: 1.49±0.47N/m^2^ vs. 1.38±0.49N/m^2^, p=0.99, mean: 0.76±0.2N/m^2^ vs. 0.74±0.18N/m^2^, p=1.00) or posterior AAo (maximum: 1.45±0.42N/m^2^ vs. 1.39±0.58N/m^2^, p=1.00; mean: 0.65±0.16N/m^2^ vs. 0.63±0.16N/m^2^, p=1.00). Both patient groups had higher maximum and mean WSS relative to the control group (p=0.001 to p=0.04). AAo peak velocity was elevated in patients compared to controls (p<0.01) but not significantly different for BB+ vs. BB- groups (p=0.42). Linear models identified significant relationships between aortic stenosis severity and increased maximum WSS (β=0.186, p=0.007) and between diameter at the sinus of Valsalva and reduced mean WSS (β=-0.151, p=0.045).

## Conclusions

BB therapy does not reduce systolic WSS or peak velocity and does not impact WSS asymmetry in the AAo of right-left fusion BAV patients. Further longitudinal studies are needed to clarify the impact of dose and duration of BB therapy on aortopathy.

## Funding

NIH NCI 5R25CA132822-04, NIH NHLBI R01HL115828; AHA13SDG14360004, BAV Program at the Bluhm Cardiovascular Institute.

**Figure 1:**
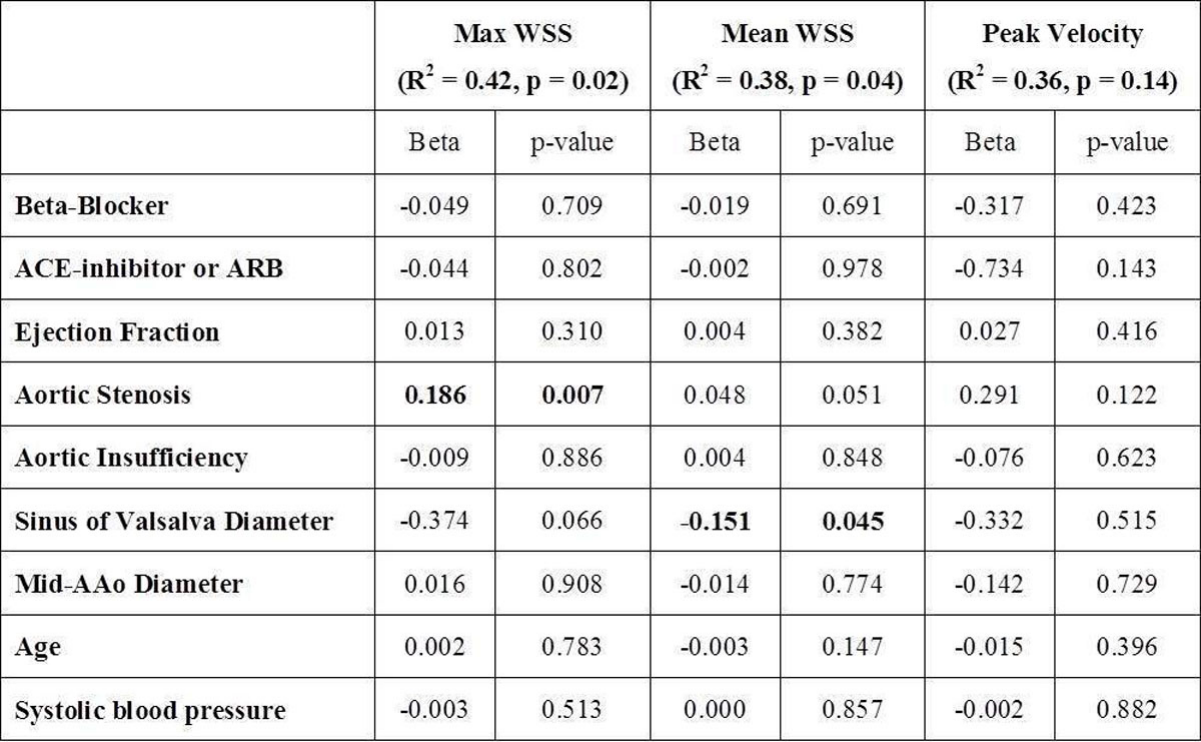
Linear Modeling Findings. AAo = ascending aorta; ACE = angiotensin converting enzyme; ARB = angiotensin receptor blocker; WSS = wall shear stress

**Figure 2:**
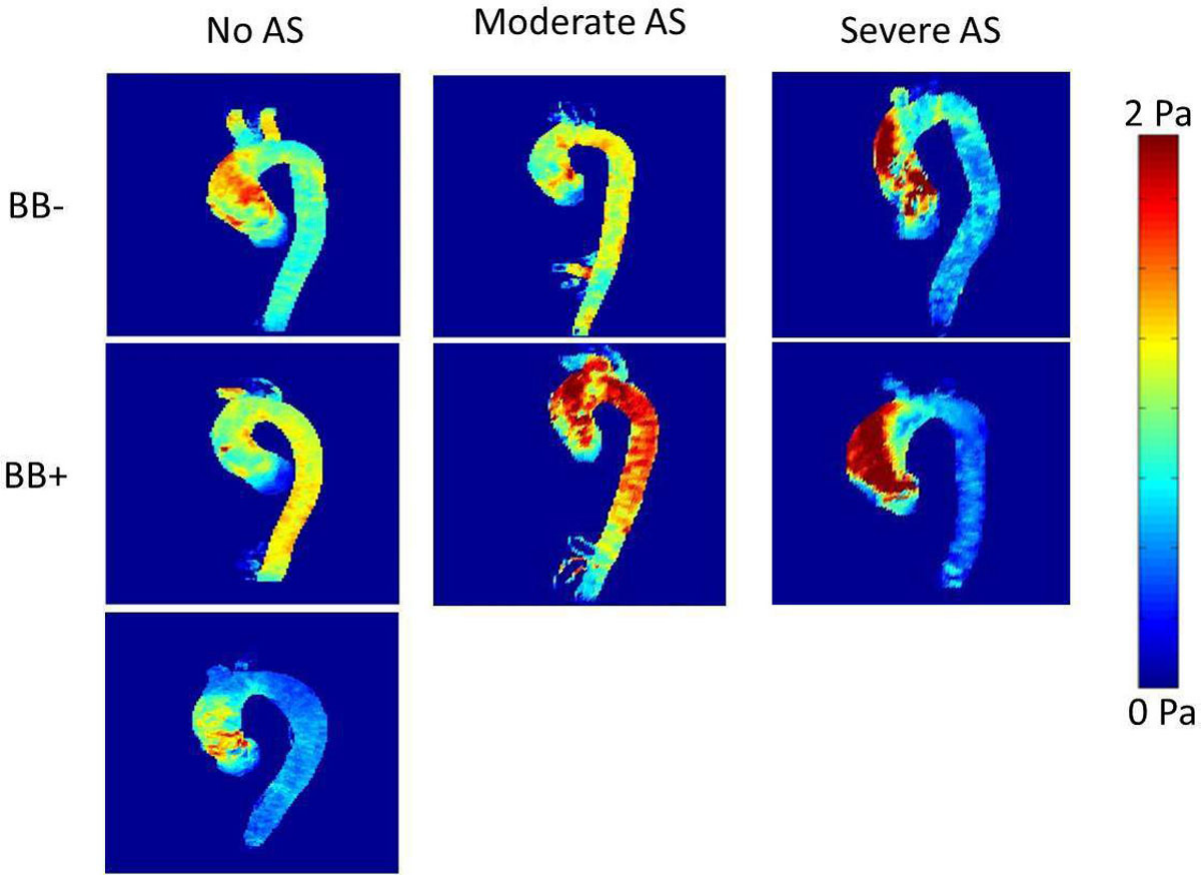
Systolic wall shear stress (WSS) maximum intensity projections (MIPs) in BAV patients with no aortic stenosis (AS), moderate AS, and severe AS from the BB+ and BB- group compared to a representative control subject.

